# A review on integrated machine learning and deep learning driven artificial intelligence models for pharmacokinetics and toxicokinetics predictions, and their application

**DOI:** 10.1016/j.dmd.2026.100240

**Published:** 2026-01-22

**Authors:** Malarvannan M, Monohar S, Sanskruti Sitaram Kate, Isha Taneja, Swati Jaiswal, Bhupesh Pratap, Prakash C. Rathi, Shikha Thakur, David Paul, Muhammad Wahajuddin

**Affiliations:** 1 Department of Pharmaceutical Analysis, National Institute of Pharmaceutical Education and Research (NIPER)-Kolkata, West Bengal, India; 2 Translational PK/PD and Investigative Toxicology, Johnson & Johnson Innovative Medicine, Spring House, Pennsylvania; 3 Certara Predictive Technologies, Simcyp Division, Certara Inc, Radnor, Pennsylvania; 4 Research and Development, Rosemont Pharmaceuticals, Leeds, United Kingdom; 5 Augmented DMTA Platform, R&D IT, AstraZeneca, The Discovery Centre (DISC), Cambridge, United Kingdom; 6 School of Medical Science and Technology, Indian Institute of Technology (IIT) Kharagpur, Kharagpur, West Bengal, India; 7 Institute of Cancer Therapeutics, School of Pharmacy and Medical Sciences, Faculty of Life Sciences, University of Bradford, Bradford, United Kingdom

**Keywords:** Hybrid learning model, Machine learning, Artificial intelligence, Toxicity, Meta-learner model, Decision trees

## Abstract

The development of artificial intelligence (AI) tools and technology has made AI-driven drug discovery a more prominent field. We are firmly in the AI era, with hybrid designs that eventually comprise deep learning (DL) and conventional machine learning (ML). Although traditional models can predict ADMET (Absorption, Distribution, Metabolism, Excretion, and Toxicity) properties, they remain relatively unsuccessful, and improving the accuracy of predictions remains challenging. Recently, several researchers have developed a hybrid learning model that successfully addresses these problems and improves prediction accuracy. The systematic tendencies facing AI-powered transformation from conventional DL and ML to hybrid learning AI models are examined in this review. Compared with traditional ML and DL, hybrid AI models have increased efficiency by reducing drug development time and costs, and improved success rates. In this context, the ongoing development of new ADMET software based on hybrid AI and multimodeling techniques can enhance the accuracy of pharmacokinetic-pharmacodynamic predictions, improve ADMET endpoint predictions, and expedite the drug discovery of new chemical entities. Moreover, this review covers the future of AI in pharmaceutical sciences and ADMET predictions, including AI-driven prediction models that range from basic ML/DL to newly developed hybrid models, evaluation parameters, and their applications in ADMET property prediction.

**Significance statement:**

The article covers the compilation of ongoing research in the development of ADMET (Absorption, Distribution, Metabolism, Excretion, and Toxicity) software based on hybrid artificial intelligence and multimodeling techniques, which may increase the accuracy of pharmacokinetic-pharmacodynamic predictions, improve ADMET endpoint predictions, and accelerate drug discovery.

## Graphical abstract

**Figure:**
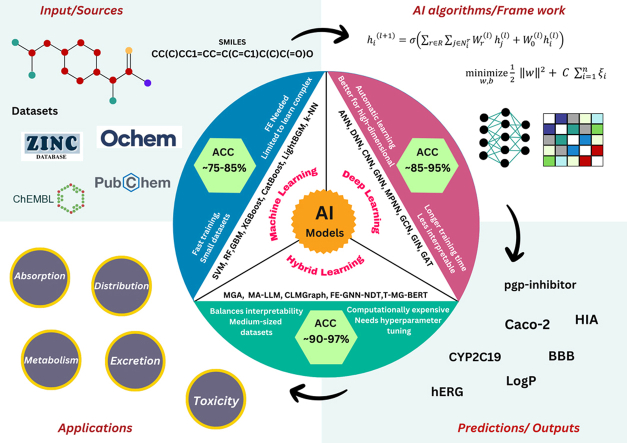

## 1 Introduction

**Fig. 1:**
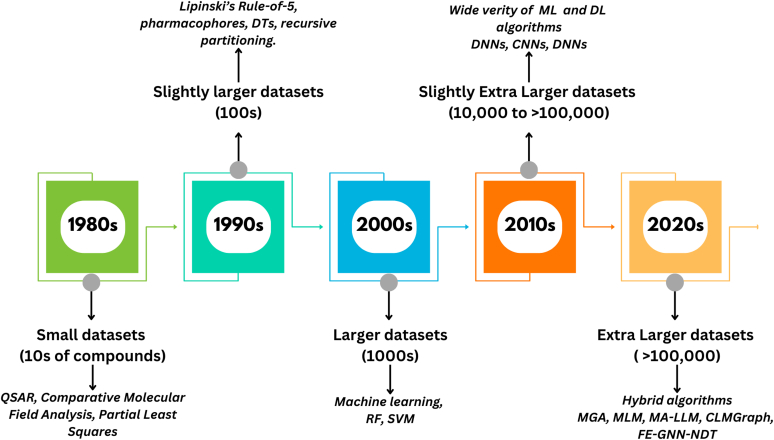
Progress of in silico ADMET predictions.

The pharmacokinetic-pharmacodynamics (PK-PD) and safety of the drug candidate, commonly referred to as ADMET (Absorption, Distribution, Metabolism, Excretion, and Toxicity) characteristics in the human body, are key parameters in the new drug development.^1^ ADMET screening is essential in pharmaceutical research because it helps identify potential drug candidates with favorable PK-PD characteristics while minimizing toxicity. Since poor ADMET profiles are a primary cause of clinical trial drug failure, precise in silico ADMET predictions are crucial to contemporary drug research.^2^ Conventional experimental approaches for evaluating ADMET, such as in vitro cell-based assays and in vivo animal studies, are costly, time consuming, and morally problematic.^3^ Nowadays, artificial intelligence (AI), which includes a range of computational methods like machine learning (ML) and deep learning (DL), can be applied across the drug development process.^4^ In the present scenario, it is simple to predict several ADMET endpoints using traditional ML/DL models, which are ultimately cost effective, provide quick predictions, and allow for scalable changes. However, new AI systems can be developed with automated decision-making capabilities. The development of in silico ADMET models has advanced significantly during the past 50 years (Fig. 1). AI-driven models can accurately predict ADMET properties by utilizing extensive chemical and biological information, significantly decreasing drug development expenses and increasing success rates.^5^ These models predict the drug’s ADMET by extracting important molecular properties and applying statistical learning. Despite their achievements, conventional ML models struggle with feature engineering and may overlook intricate nonlinear relationships between ADMET characteristics and molecular structures. To overcome this limitation, DL models, which can automatically learn hierarchical molecular characteristics from raw data, have become popular.

The accuracy of ADMET models has significantly increased according to DL; however, interpretability and computational efficiency remain issues that necessitate a shift toward hybrid learning strategies. Hybrid learning models combine the advantages of many approaches, such as ML, DL, and multimodal approaches, to improve ADMET predictions.^6^ Comparing these models with ML/DL models alone, they are intended to manage diverse datasets, increase prediction accuracy, and provide superior interpretability. There are various types of hybrid ADMET learning models, which include ML-DL hybrids (ML for feature selection and DL for complex pattern recognition), multimodal hybrids (that combine fingerprints, molecular descriptors, SMILES (Simplified Molecular Input Line Entry System), and structural data for robust predictions), ensemble learning (stacking of multiple ML/DL models to improve predictions and reduce bias), and transfer learning (application of previously trained DL models to new ADMET tasks to improve generalization) models.^7^ By accelerating drug development, these hybrid models help pharmaceutical companies find possible therapeutic candidates with superior ADMET properties and screen compounds more effectively. With the shift from simple ML/DL models to hybrid AI-driven models, ADMET prediction enters a new phase.^8^ Incorporating ML, DL, and hybrid approaches allows researchers to make predictions more quickly, accurately, and economically, ultimately speeding up drug discovery and providing better interventions for better human health.

In this context, the ongoing development of new ADMET software based on hybrid AI and multimodeling techniques can increase the accuracy of PK-PD predictions, improve ADMET endpoint predictions, and accelerate the discovery of new chemical entities. Moreover, the future of AI in pharmaceutical sciences and drug discovery is covered in this review, along with the use of AI-driven prediction models, ranging from basic ML/DL to newly developed hybrid models.

## 2 Metrics in AI prediction evaluation

Metrics play an important role for ADMET prediction because they evaluate model performance and assure accuracy as well as reliability of the outcomes. They are helping to find the potential drugs and minimizing the possibility of significant failures. Various metrics are used to evaluate the accuracy, efficiency, and dependability of AI models, especially when it comes to prediction tasks. Depending on the kind of prediction task, these metrics can be roughly divided into 2 categories: regression metrics and classification metrics. When the output is continuous, regression metrics are used to predict numerical values based on the input features. This can aid in measuring the accuracy and reliability of the ADMET model's prediction. On the other hand, the classification matrix is employed in the ADMET when the outcome is categorical for instance; for example, classification of toxic versus nontoxic. Binary and multiclass classifications are 2 other categories that are useful for determining how well the ADMET model predicts outcomes. For example, a single fixed threshold is used to calculate accuracy (ACC), precision (PR), sensitivity (Recall), and associated metrics, and these values changed when the threshold change. The list of evaluation metrics and their formulas is presented in Table 1. All of these metrics aided in improving the model architecture by assessing the model’s performance and comparing it to other AI models.^9^

**Table 1:** List of evaluation metrics for AI model performance and its formulas

Parameters	Class	Formula	Outcome/ Indicates	Equation Number
Coefficient of determination (R^2^)	Regression	$R^{2}=1-\frac{\sum {\left(y_{i}-ŷ\right)}^{2}}{\sum {\left(y_{i}-ӯ\right)}^{2}}\text{Range}\;\left[0,1\right]$	The R2 score, which measures the proportion of dependent variables that are predictable from independent variables, is also known as the coefficient determination. The higher value range between 0 and 1 indicates better model performance.	1
Root mean squared error (RMSE)	Regression	$\text{RMSE}=\sqrt{\frac{1}{N}\sum_{i=1}^{N}{\left(y_{i}-ŷ\right)}^{2}}$	The square root of the MSEMSE represents the RMSE. It is easier to interpret because it gives an error measure in the same units as the target variable.	2
Specificity (SP)	Classification	$\text{SP}=\frac{\text{TN}}{\text{TN}+\text{FP}}\text{Range}\;\left[0,1\right]$	SP is the capacity to accurately recognize negative outcomes. Lower specificity values correspond to higher FP rates, reflecting an increased misclassification of negative cases as positive. A specificity value of 1 indicates that all negative cases are correctly identified, whereas a value of 0 indicates that none of the negative cases are correctly classified.	3
Matthew’s correlation coefficient (MCC)	Classification	$\text{MCC}=\frac{\text{TP}.\text{TN}-\text{FP}.\text{FN}}{\sqrt{\left(\text{TP}+\text{FN}\right).\left(\text{TP}+\text{FP}\right).\left(\text{TN}+\text{FN}\right).\left(\text{TN}+\text{FP}\right)}}$	The quality of binary classifications is assessed using MCC. Even with the unbalanced datasets, the evaluation matrix is balanced. MCC provides a single figure that sums together performance for every class.	4
Precision (PR)	Classification	$\mathrm{PR}=\frac{\text{TP}}{\text{TP}+\text{FP}}\text{Range}\;\left[0,1\right]$	PR measures the proportion of TPs among all positive predictions. The fewer false alarms, the higher the PR. utilized it in situations where precise positive predictions are to be accurate.	5
Accuracy (ACC)	Classification	$\text{ACC}=\frac{\text{TP}+\text{TN}}{\text{TP}+\text{TN}+\text{FP}+\text{FN}}\text{Range}\;\left[0,1\right]$	Overall proportion of accurate predictions, both positive and negative. With zero FPs and zero FNs, a perfect model would have an accuracy of 1.	6
Jaccard	Classification	$\text{Jaccard}=\frac{\text{TP}}{\text{TP}+\text{FP}+\text{FN}}\text{Range}\;\left[0,1\right]$	Measures similarity between predicted and actual positives. The dataset is the same, and the outcome is 1. If 0, there are no common elements among the datasets.	7
Recall (Sensitivity)	Classification	$\text{Recall}=\frac{\text{TP}}{\text{TP}+\text{FN}}\text{Range}\;\left[0,1\right]$	The proportion of accurately predicted positive instances to all actual positive instances is known as recall. It measures how well all relevant positive cases are captured by the model. The values 1 indicates perfect recall and 0 indicates the model has the worst recall, failing to detect any real positive.	8
F1 score	Classification	$F1\;\text{score}=\frac{2\times \text{Precision}\times \text{Recall}\;}{\text{Precision}+\text{Recall}\;}$	Recall and precision are combined calculation is F1 score; this is helpful for datasets that are unbalanced. There is no prediction error when the F1 score is 1.	9
AUC	Classification	---	Evaluates the classifier's capacity for class distinction. The likelihood that a classifier would rank a randomly selected positive example higher than a negative example is known as the classifier's AUC. AUC is the integral of the true positive rate with respect to the false positive rate from 0 to 1.	10

The total number of observations, the actual value for the ith observation, the predicted value of y, and the average value of y are denoted by N, $y_{i}$, ŷ, and ӯ in equations 1 and 2, respectively. The metric's inputs in equations 3–9 are the numbers of true negatives (TN), false negatives (FN), true positives (TP), and false positives (FP).

## 3 Types of models for ADMET predictions

**Fig. 2:**
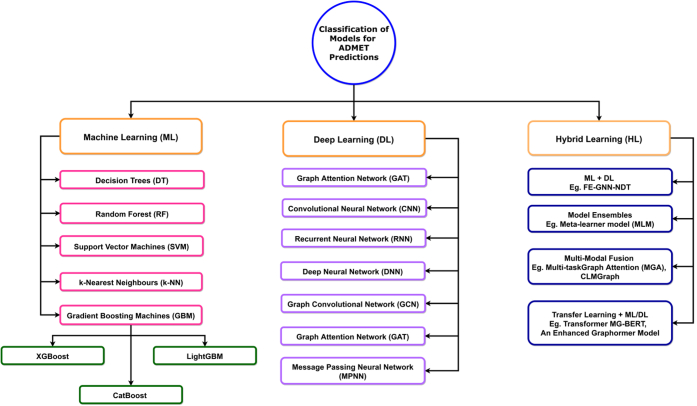
Classification of AI prediction models of ADMET.

In order to assess PK and safety characteristics at an early stage of development, ADMET predictions are essential in drug discovery. For ADMET predictions, a number of computational models have been developed; these can be generally classified as ML-based, DL-based, and hybrid learning-based models. With regard to accuracy, interpretability, and data requirements, each model type has unique advantages. A brief classification and overview of these model types are illustrated in Fig. 2 and discussed below.

### 3.1 ML models

ML models, which are computational techniques that find patterns in data, can be used to make ADMET predictions or decisions without the need for explicit programming. These models are classified into 2 categories: supervised and unsupervised learning methods. A supervised learning model is trained by using labeled data, where input-output pairs direct the learning process. These models include regression (eg, Linear Regression and Random Forest [RF]) and classification (eg, Decision Trees, support vector machines (SVMs), and neural networks (NNs)). Whereas unsupervised learning models are trained by using unlabeled data such as uncover hidden structures. For example, these models include dimensionality reduction methods like pincipal component analysis and t-distributed stochastic neighbor embedding that aid in the visualization of high-dimensional data, and clustering algorithms like K-Means provide groups of comparable data points. The typical uses of this model are customer segmentation, anomaly detection, and feature extraction for the prediction process.

#### 3.1.1 Random forest

An ensemble learning approach called RF uses several decision trees to decrease overfitting and increase prediction accuracy. It applies to regression and classification tasks using averaging, random feature selection, bagging (boot aggregations), and classification to provide predictions.^10^ For instance, Svetnik et al^11^ used the RF to examine its accuracy by using 6 QSAR datasets which include 4 classifications (blood-brain barrier [BBB], estrogen, P-glycoprotein, and multidrug resistance reversal) and 2 regression datasets (COX-2 and dopamine), and the accuracy studies found that it provides more than 80% accuracy in the 50 replications of 5-fold cross-validation. The results found that it is beneficial to aggregate classifiers that start out with low bias.^11^ Similarly, in developing the ADMET prediction model, Lei et al^12^ developed a quantitative and qualitative predictive model for urinary tract toxicity using the recursive feature elimination approach combined with RF for dimension reduction. They have employed this method, along with 6 additional ML models. Comparing the 10-fold cross-validated RF model for this study with others, the results demonstrated better performance.^12^

#### 3.1.2 Gradient boosting machines

Multiple decision trees are repeatedly combined into a single, more effective prediction using gradient boosting machine (GBM) prediction models to produce a high performance ensemble by compensating for the errors from the preceding stage. Several GBM algorithms have been developed, including XGBoost, LightGBM, and CatBoost. Each different GBM algorithm has a distinct decision tree structure, with some modifications to the original structural model. In ADMET property prediction, XGBoost, LightGBM, and CatBoost are frequently utilized.^13^ With its strong feature selection and ability to handle unbalanced datasets in toxicity prediction, XGBoost is effective for structured data. LightGBM employs leaf-wise growth to enhance drug likeness classification and is optimized for speed and large-scale bioinformatics data analysis. CatBoost reduces preprocessing requirements in PK modeling by excelling at handling categorical features. However, mathematical additive model for a GBM, firstly, the function f(x), which maps the relationship between inputs like chemical descriptors and biological activity ($x_{i}$, $y_{i\;}$ respectively), is the basis of the gradient boosting methods. The function is expressed in eq. 11.

(11) $$\hat{F}\left(x\right)=\sum_{m=1}^{M}\sigma *\hat{F_{m}}\left(x\right)$$

$\hat{F_{m}}\left(x\right)$ is the mth tree, andσ is the learning rate. Using a function loss $L\left(y_{i},p_{i}\right)$ like the binary cross-entropy, which quantifies the quality of prediction $p_{i}$ about actual readouts $y_{i}$, each tree $\hat{F_{m}}$ is learned by reducing the following goals after the initial iteration.

(12) $$\hat{F_{m}}=argminE\left(\frac{-\partial L\left(Y,P_{m-1}\right)}{\partial P_{m-1}}-P_{m}\right)$$

where the current iteration prediction is ${\;P}_{m}$. The loss function L compares actual values Y and predictions $P_{m-1}$ from the previous model. As a result, every new decision tree is built to make up for the model's prediction errors from the prior iteration. This is simply a gradient descent in a function space rather than a parameter space.^14^

These mathematical additive models of GBM are applied to several predictions of ADMET properties. For instance, An et al^15^ applied GBM using 3 evaluation metrics, including mean squared error (MSE) and R2, to predict ERα bioactivity and ADMET properties. The results showed that GBM with RF ensembles improved R^2^ scores for regression problems, achieving values above 0.7, indicating accurate and dependable fits for bioactivity predictions. Similarly, ADMET prediction shows high performance on external validation sets with accuracies ranging from 0.76 to 0.96 and F1 scores from 0.80 to 0.97.^15^

#### 3.1.3 Extreme gradient boosting

Extreme gradient boosting (XGBoost) is a powerful algorithm built on gradient boosting methods. It is frequently utilized in tasks involving supervised learning, especially when it comes to prediction, regression, and classification. This model improves performance by using an ensemble of sequentially trained decision tree models. The optimization of the objective function, which combines a regularization term and a loss function, forms the basis of the XGBoost mathematical equation.^16^

If D is the training set consisting of m features and n labels, the decision tree in the XGBoost model makes the predictions for the sample.

(13) $$D=\left\{\left(x_{i},y_{i}\right)\left(D=n,x_{1}\in R^{m},y_{i}\in R^{n}\right)\right\}$$

$g_{j}\left(x_{i}\right)=w_{q}\left(x_{i}\right)$ is used by the model for $j^{th}\text{.}$ A decision tree to predict the sample$\left(x_{i},y_{i}\right)\text{.}$Equation 14 is used to calculate the sum of all M decision tree predictions in the XGBoost model.

(14) $${ŷ}_{i}=\sum_{j=1}^{M}g_{j}\left(x_{i}\right)$$

where $w_{q}$ is the leaf weights. Optimizing the objective functions will reduce overfitting in the XGBoost model, which consists of a loss function and a regularization term.

(15) $$obj\left(\theta \right)=\sum_{i=1}^{N}l\left(y_{i},{ŷ}_{i}\right)+\sum_{j=1}^{M}\Omega \left(f_{i}\right)$$

where $\Omega \left(f_{i}\right)=\gamma T+\frac{\lambda }{2}\sum_{l=1}^{T}w_{l}^{2}$ is the regularization term, T denotes number of leaves, and λ,γ are the regularization parameters.^17^

Based on the output of the previous decision tree, XGBoost interactively trains a new tree during training and then approximates the objective function's first and second derivatives using a Taylor expansion, which can be expressed as follows.

(16) $${Obj}^{\left(t\right)}\approx \sum_{i=1}^{N}\left[g_{i}f_{t}\left(x_{i}\right)+\frac{1}{2}h_{i}f_{t}^{2}\left(x_{i}\right)\right]+\Omega \left(f_{i}\right)$$

where $g_{i}=\frac{\partial l\left(y_{i},{ŷ}_{i}^{\left(t-1\right)}\right)\;}{\partial {ŷ}_{i}^{\left(t-1\right)}}$ is the first-order gradient and $h_{i}=\frac{\partial^{2}l\left(y_{i},{ŷ}_{i}^{\left(t-1\right)}\right)}{\partial {ŷ}_{i}^{{\left(t-1\right)}^{2}}}$ is the second-order gradient (Hessian).

For the ADMET endpoint prediction, Wu et al^18^ effectively used those mathematical additive models of XGBoost. On the same benchmark datasets, XGBoost outperformed alternative algorithms by achieving the key ADMET prediction accuracies in this study, which included Caco-2 (94.0%), CYP3A4 (95.7%), human ether-à-go-go-related gene (hERG) (89.4%), hepatotoxicity (88.6%), and mutagenicity (96.2%).^18^ Similarly, Li et al^19^ utilized the same XGBoost algorithms to directly predict the maximum daily dose of a compound using existing human research data. It attained extremely high regression accuracy, demonstrating XGBoost's effectiveness for ADMET modeling's classification and regression endpoints.

#### 3.1.4 Support vector machines

SVM determines the best hyperplane for separating molecular descriptors into several classes of ADMET properties. SVM is utilized in ADMET prediction because of its capacity to manage complex relationships and high-dimensional data.^20^ Finding the hyperplane that divides the data points into distinct classes while optimizing the margin between them is the goal of the SVM method.^21^ Equations (17), (18), (19), (20) are the mathematical expressions for this.

The given training set is $\left(x_{i},y_{i}\right)$. where $x_{i}$ are input vectors such as physical and chemical properties of molecules, and $y_{i}$ are the associated label which represents the output (target) variable assigned to each molecule. The SVM decision function is calculated using eq. 13, followed by the optimization of the problem using eq. 17

(17) $$f\left(x\right)=w^{T}x+b$$

(18) $$\underset{\;w,b}{\text{minimize}\;}\frac{1}{2}{\left\|w\right\|}^{2}+C\sum_{i=1}^{n}\xi_{i}$$

Subject to $y_{i}\;\left(w^{T}x+b\right)\geq 1-\xi_{i}$ , $\xi_{i}\geq 0$, where w is the normal vector to the hyperplane, b is a bias term, C is the regularization parameter, and $\xi_{i}$ is a slack variable.

Later, the kernel SVM is applied to find the nonlinear relationships of the given dataset by using radial basis function (eq. 19).

(19) $$K\left(x_{i},x_{j}\right)=\exp\;\left(-\gamma {\left\|x_{i}-x_{j}\right\|}^{2}\right)$$

The kernel is a function K, such that for each x,zϵX

(20) K(x,z)=⟨ϕ(x).ϕ(z)⟩

where ϕ is a mapping of X to a feature space, feature vector x or z consists of numerical values corresponding to different properties of a molecule. Finally, the prediction was given after this training, and the decision function for a new sample x is given by eq. 21, where $\alpha_{i}$ language multipliers obtained during training.^22^

(21) $$f\left(x\right)=\sum_{i=1}^{n}\alpha_{i}y_{i}\langle \phi \left(x_{i}\right).\phi \left(x\right)\rangle +b$$

In application to predict ADMET, Ogura et al^23^ successfully used the SVM model for predicting hERG inhibitory activities. Their fingerprints recorded kappa statistics of 0.733 and accuracy of 0.984 for the test set, significantly outperforming the performance of the hERG prediction applications. Similarly, the study by Yang et al^24^ investigated several ML algorithms for the accuracy of the ADMET evaluation, often placing SVM as one of the top performers. For instance, on certain datasets, cubic SVM achieved classification accuracy of 95.5%, which significantly reduces the effort required for selection and optimization of ADMET properties model algorithms.^24^

#### 3.1.5 k-nearest neighbors

The k-nearest neighbors (k-NN) algorithm is a simple, nonparametric ML method used for both regression and classification. The majority class of a new data point's k-NN in the feature space is used to classify it. This approach is frequently utilized in QSAR models. The foundation of this approach is the idea that molecules with similar structures exhibit similar functions. Weighing the contributions of neighbors is reasonable, though. For example, a similar structure with similar biological activity also has identical activity, indicating that near neighbors contribute more to the projected value. The k-NN approach does not predict if none of the nearest neighbors satisfy the condition.^25^

The similarity distance between the 2 small molecules is important in this case. This can be calculated using the widely used Tanimoto distance, d, defined as follows:

(22) $$d=1-\frac{n\left(P\cap Q\right)}{n\left(P\right)+n\left(Q\right)-n\left(P\cap Q\right)}$$

The total number of features is n(P) and n(Q), where n(P∩Q) is the number of features that both p and q share. The molecular similarity is calculated using these features. Depending on the problem and dataset, additional distance calculations are used, such as the Manhattan and Euclidean distances.^26^

The predicted activity *y* is then given by the average across structurally similar neighbors

(23) $$y=\frac{\sum_{i=1}^{v}y_{i}e^{-{\left(\frac{d_{i}}{h}\right)}^{2}}}{\sum_{i=1}^{v}e^{-{\left(\frac{d_{i}}{h}\right)}^{2}}},d_{i}\leq d_{0}$$

where ${\;d}_{0}$ is a Tanimoto distance threshold, ${\;d}_{i}$ is the Tanimoto distance between molecule and the molecule i of the training set, $y_{i}$ is experimental measure of molecule i, h is the smoothing factor, and v is the total number of molecules in the training set that satisfy the condition $d_{i}\leq d_{0}$.

The k-NN algorithm has been successfully utilized by several researchers for ADMET endpoint predictions, yielding reasonable results when compared to other models. To address the issue of long prediction times in previous computation models, Schyman et al^27^ developed the ADMET prediction model by utilizing the k-NN algorithm, which involves the different ADMET predictions, such as metabolic stability, and various toxicity risks, such as hepatotoxicity and hERG inhibition, and yields quantitative accuracy that is closer to the outcomes of intricate ensemble approaches. Additionally, Uddin et al^28^ compared the k-NN algorithm for disease prediction, finding that ensemble and adaptive variations frequently outperformed the regular k-NN, with classification accuracies ranging from 64% to 84%.

### 3.2 DL models

DL is a subset of ML that uses multiple layered NNs to learn complex, nonlinear relationship directly from the data which are based on linear regression followed by some activation functions. Unlike traditional statistical linear regression, DL consists of many neural nodes instead of only one node. Neural units in a layer can range from hundreds or even thousands. The nodes are referred to as hidden nodes, whereas the layers that lie between the input and the output are referred to as hidden layers.^29^ Similarly, unlike traditional QSAR models, DL models automatically learn hierarchical representations from raw inputs like chemical structures, molecular graphs, or biological descriptors, in contrast to conventional ML techniques that mostly rely on manually created features. Activation function is another important factor in the DL which is inspired by the human neural firing, ie, it either fire or not, which is used to generate the nonlinear relationships between the input and outputs. There are many activation functions which combined with the many neural nodes and layer and mimics the human brain like structure. The activation functions include sigmoid, Hyperbolic tangent, and rectified linear unit (ReLU). The activation function is responsible for transforming the data onto a different plane so that the impacts of various dimensions in the given problem may be observed. In most cases, the data are tightly clustered.^30^

**Fig. 3:**
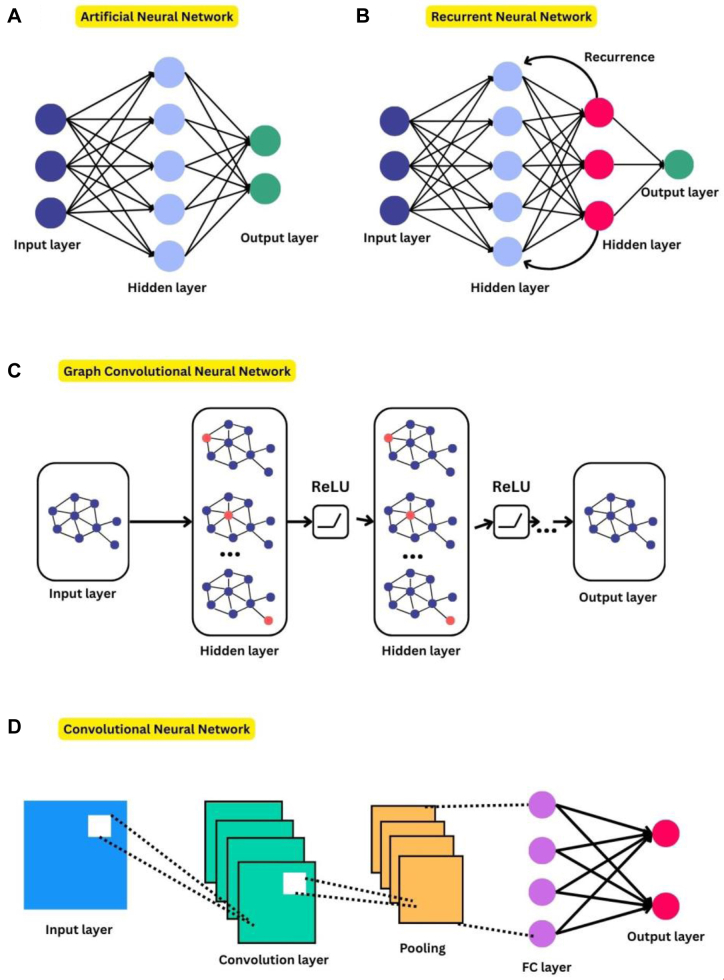
Architectures of DL models. (A) ANNs. (B) RNNs. (C) Graph convolutional neural networks. (D) CNNs.

Building on these principles, DL has revolutionized ADMET prediction by enabling accurate drug screening through the capture of complex molecular patterns. Based on NN methods, a number of DL models are developed to assist ADMET predictions. The models (Fig. 3) artificial neural networks (ANNs), convolution neural networks (CNNs), recurrent neural networks (RNNs), and message passing neural networks (MPNNs) each have distinct layers and structures and are used for various ADMET prediction applications. For example, Fralish et al^31^ developed DeepDelta, a DL model that uses MPNN algorithms to improve chemical derivatives and predict ADMET. Similarly, Cáceres et al^32^ described how deep neural network (DNN) algorithms function in ADMET predictions, consistently generating further datasets and increasing computational capacity.

#### 3.2.1 Artificial neural networks

ANNs are based on the human brain and nervous system. Specifically ANN models stimulate the electrical activities of the brain and nervous system. The processing elements which include neurodes are connected with other processing elements such as layers or vectors. These connections simulating the synaptic connections of the brain simulate the electrical excitation of a nerve cell and consequently the transference of information within the network or brain. The outputs are stimulated by multiplying the inputs $i_{n}$ by a connecting weight $w_{n,m}$. To generate a single input value for the neurode, all of the weight-adjusted input values to a processing element are then combined using a vector-to-scalar function such as summation ($y=\sum w_{ij}x_{i}$), averaging, input maximum, or mode value. The processing components employ the estimated input to transform the function and generate outputs.^33^

ANNs comprise interconnected layers of neurons, with input, hidden, and output layers being the primary components. Nodes, or neurons coupled to one another, make up each layer. ANNs are self-learning models that make predictions only by utilizing their understanding of SMILES code. Furthermore, the ANN aids in the prediction of the target compounds hepatotoxicity, permeability, and solubility.^34^ ANN can predict the BBB permeability of any drug. For example, Guerra et al^35^ developed the ADMET model using the ANN algorithm to predict BBB penetration. The model achieved an average accuracy of 83% in the training set and 73% in the external prediction set.

#### 3.2.2 Convolutional neural networks

Unlike traditional ANNs, CNNs perform classification by leveraging contextual and spatial information present in the input data. The CNN architecture comprises input, convolutional, pooling, activation function, fully connected (FC), and output layers. The stacked convolution layer can detect local conjunctions of features. The receptive field, which is linked to some neurons in the previous layer, is used to extract the local features of inputs. A neuron's receptive field linked to a particular region in the previous layer forms a weight vector that is constant throughout the plane, which is made up of the neurons in the subsequent layer. The feature map is generated by sliding the weight vector over the input vector while the neurons in the plane share the same weights. Convolution operation is the process of moving the filter both horizontally and vertically. Equation 24 provides the mathematical expression of this operation getting out put ($a_{ij}$).^36^

(24) $$a_{ij}=\sigma {\left(W*X\right)}_{ij}+b$$

where X is the input feature map, W is the convolutional kernel applied across the input, b represents the bias, **∗** denotes the convolution operator, and σ is the activation function that introduces nonlinearity into the model.

The pooling layer helps mitigate overfitting issues which reduces number of trainable parameters and introduces translation invariance. It creates a nonlinear downsampling, and the FC layer learns the various features and their contributions to the outputs. CNNs predominantly employ the ReLU activation function rather than the sigmoid function commonly used in traditional ML models. This preference is due to several advantages: first, ReLU and its derivative are computationally simple and efficient, second saturating nonlinearities such as the sigmoid function tend to slow down training, whereas nonsaturating functions like ReLU enable faster convergence. Additionally, ReLU helps mitigate the vanishing gradient problem. CNN is used extensively in image processing and is also applied to ADMET predictions, specifically analyzing fingerprints, SMILES, and molecular structures.^37^ The CNN is frequently implemented in ADMET predictions due to its lower complexity when compared with other NNs. Tran et al,^38^ for example, developed the CNN-based Ames mutagenicity prediction model AMPred-CNN, and the same methodology was used by Shi et al,^39^ for ADMET prediction using the CNN method based on a 2D molecular image, successfully extracting critical molecular aspects of the inputs' ADMET properties.

#### 3.2.3 Other NNs

The ADMET predictions also use several other NN models, including graph neural networks (GNNs), MPNNs, and graph convolutional networks (GCNs). Due to their exceptional ability to simulate the intrinsic graph-like structure of molecules, in which atoms serve as nodes and chemical bonds as edges, GNNs have gained significant recognition in molecular property prediction. In contrast to other DL techniques that use a one-dimensional chemical description to predict ADMET properties, GNNs work with graph-structured data. They can capture complex, multiscale molecular interactions. This aids in figuring out the target molecules' biological activity and other characteristics.^40^ The fundamental idea of MPNN is to enhance the prediction of molecular properties while GNN is in operation. By aggregating and updating node attributes based on nearby nodes, MPNNs iteratively spread information throughout the molecular network. The model learns hierarchical representations through several layers of message forwarding, which enable it to recognize intricate patterns in molecular data, including stereochemistry, aromaticity, and ring structures.^41^ Additionally, certain models are developed based on GNN architecture. For instance, GCNs excel at capturing local structural elements because they employ convolution operations on graph data. Graph attention networks, on the other hand, use attention processes to prioritize particular atomic interactions that have a major influence on molecular properties by weighing neighboring nodes.^39^ The Graph Isomorphism Networks (GINs), which improve predictions, emerged recently. Enhanced GIN predicts ADMET qualities end to end by taking advantage of bond features and the atom differences.^42^

### 3.3 Hybrid learning models

ML and DL are combined in hybrid learning models to improve ADMET prediction. Hybrid models improve accuracy and generalization by capturing intricate molecular interactions through the integration of many techniques. Recently, several hybrid learning models have been developed and effectively applied to enhance the ADMET model which are detailed in this section.

#### 3.3.1 Multitask graph attention

**Fig. 4:**
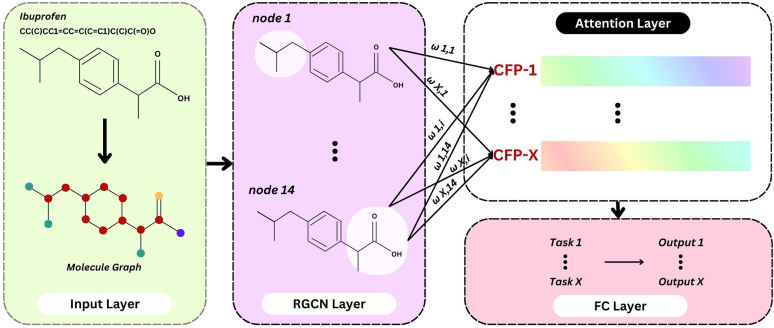
MGA model architecture.

Multitask graph attention (MGA) is an advanced graph-based multitask learning model that uses graph attention processes to predict ADMET properties and extract features. The input layer, relation graph convolutional network (RGCN) layers, attention layer, and FC layers comprise the 4 layers that build the MGA (Fig. 4). After passing through the RGCN layer, the node represents the overall characteristics of the circular substructure centered on the atom. The input is the information of the atom represented by the node.^43^ To manage graph data with various kinds of relationships (edges), RGCN operates as an extension of ordinary GCNs.^44^ They work especially well with networks that have nodes connected by several types of edges. Equation 25 determines each node's propagation.

(25) $${h_{i}}^{\left(l+1\right)}=\sigma \left(\sum_{r\in R}\sum_{j\in N_{i}^{r}}W_{r}^{\left(l\right)}h_{j}^{\left(l\right)}+W_{0}^{\left(l\right)}h_{i}^{\left(l\right)}\right)$$

where ${h_{i}}^{\left(l+1\right)}$ is the state vector of the target node i, after l+1 iterations, $N_{i}^{r}$ is set of neighbors of node i connected by relation (edge) r∈R, $W_{r}^{\left(l\right)}$ is relation-specific learnable weight matrix edge type r at layer l, $W_{0}^{\left(l\right)}$ is self-loop weight matrix or own feature of node i, and σ is nonlinear activation function. As demonstrated above, an RGCN explicitly incorporates edge information under the relation ∈R.

Once the attention layer has produced the customized fingerprints for general features, it can then assign various attention weights to the various substructures, both produced by subsequent computations, as follows.

(26) $$\omega_{i}=\sigma \left(W.\;h_{i}+bias\right)$$

(27) $$\text{Customized}\;\text{fingerprints}=\sum_{i=1}^{N}\omega_{i}.\;h_{i}$$

where $\omega_{i}$ denotes the attention weight of node (substructure) i, N is the number of nodes (substructure), $h_{i}$ is the general feature of nodes (substructure) i, and W and bias are the attention layer parameters for the model training.

Lastly, the FC layer uses the customized toxicity fingerprints to predict the corresponding task. The loss function of MGA is a combination of ${loss}_{c}$ and ${loss}_{r}\text{.}$ Finally, the output ADMET values for the compound of interest were generated.

(28) $${\text{loss}}_{c}=\sum_{i=1}^{N}\sum_{c}^{C}\left(-\left[P_{c}y_{n.c}\;.\log\;\sigma \left(x_{n.c}\right)+\left(1-y_{n.c}\right)\;.\log\left(1-\sigma \left(x_{n.c}\right)\right)\right]\right)$$

(29) $${\text{loss}}_{r}=\sum_{i=1}^{N}\sum_{r}^{R}{\left(x_{n.r}\;.\;y_{n.r}\right)}^{2}$$

(30) $$\text{loss}={loss}_{c}+{loss}_{r}$$

where ${\;x}_{n.c}$ represents the molecule n’s predicted value for the classification task c, $y_{n.c}$ represents the molecule n’s true values for the classification task c. The weight of positive samples is represented by $P_{c}$ and the predicted value of molecule n for the regression task r is represented by $x_{n.r}$. The true value of molecule n for the regression task*r* is $y_{n.r}$. The number of molecules is N, and the molecules is represented by *r*, the number of regression tasks is R and the number of classification task is C.

#### 3.3.2 Meta-learner model

**Fig. 5:**
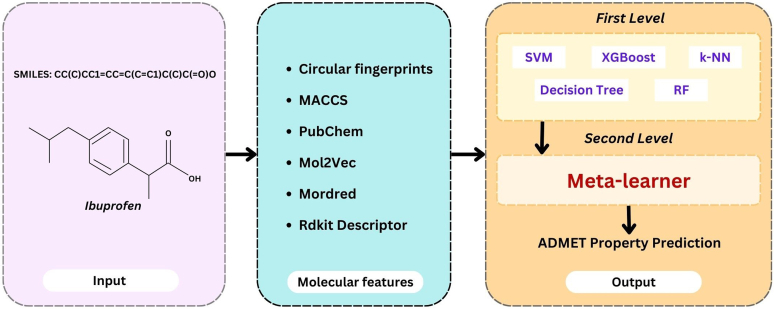
MMA architecture.

Combining multiple ML submodels, meta-learner model is a hybrid ML model. Combining predictions from several models yields powerful results. By using learning algorithms in place of the linear weighed sum that was previously used to integrate the submodels' predictions, this method goes beyond the standard. The meta-learner model can learn to effectively combine the scores from the heterogeneous models and give the better prediction of the ADMET properties. The input layer is the first layer of the model, which is followed bimolecular feature conversion and 2 learning model levels (Fig. 5). Five ML models, including SVM, RF, k-NN, decision tree, and XGBoost, are included in the first level, which uses the given information to generate a prediction. The meta-learner is the second-level linear prediction model, which acquires the ability to aggregate initial prediction scores and generate predictions appropriately. Gaining a deeper comprehension of the input molecules and increasing prediction accuracy are the responsibilities of the meta-learner. However, because of the high correlation between the different classifiers, the meta model performs better than individual classifiers. Consequently, the meta-modal performance is significantly enhanced. The meta model, on the other hand, issues with the "Half-Life" assignment due to a disagreement between the models.^45^

#### 3.3.3 Multi-agent large language model

**Fig. 6:**
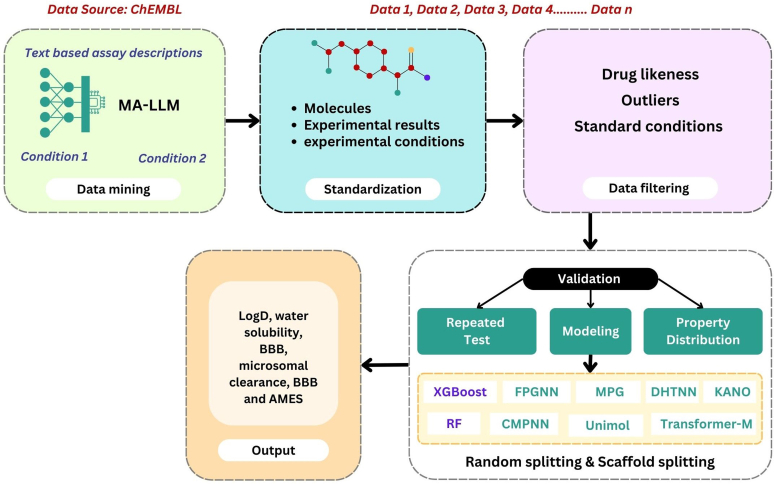
MA-LLM architecture.

The current ADMET prediction models lack compound representation and have a small dataset size. To solve this problem, a data mining system based on multi-agent large language model (MA-LLM) efficiently determines experimental conditions from bioassay data. A customized multi-agent system with varying capabilities was proposed to automate the intricate data mining procedure. This method combines entries from various sources to produce the final outcome. The MA-LLM approach effectively operates with a small number of samples, in contrast to supervised models that need thousands of data for fine-tuning. Multiple validation procedures are included in this modeling approach to verify the molecular properties, modeling capabilities, and data quality.^46^ The experimental conditions are extracted using the first MA-LLM from the assay descriptions of the primary ChEMBL database. The experimental results from different sources are then standardized in the second layer, and the data are filtered in the third layer according to factors, experimental values, and drug likeness. Lastly, for AI modeling purposes, post process the datasets by eliminating duplicate test results and separating them using the random and scaffold splitting methods (Fig. 6). Following the completion of data collection, 3 aspects of technical validation are performed to show that the workflow is improving the data quality. This approach involves DL and ML modeling, analysis of property distribution, and repetitive testing for data quality assessment. Nevertheless, 2 ML techniques XGBoost and RF as well as extended connectivity fingerprints as molecular descriptors are used in AI modeling. Additionally, 7 DL models, Communicative Message Passing Neural Network, Fully-Parameterized Graph Neural Network, Dynamic Heterogeneous Transformer Neural Network, Knowledge-Aware Neural Operator, Molecular Property Graph, Unimol, and Transformer-M, are used for model validation. The dataset is split into 2 random and scaffold splitting for this validation method. Finally, the model datasets linked to regression tasks are used to determine mean absolute error and root mean squared error and reasonably high R values, which indicate the prediction of acceptable metrics for LogD, water solubility, BBB, and microsomal clearance. However, the prediction for CYP is still comparatively low and requires additional advancements. The BBB and AMES datasets classifications are accurately predicted by the approach.^47^

#### 3.3.4 Contrastive learning-based multitask graph neural network model

**Fig. 7:**
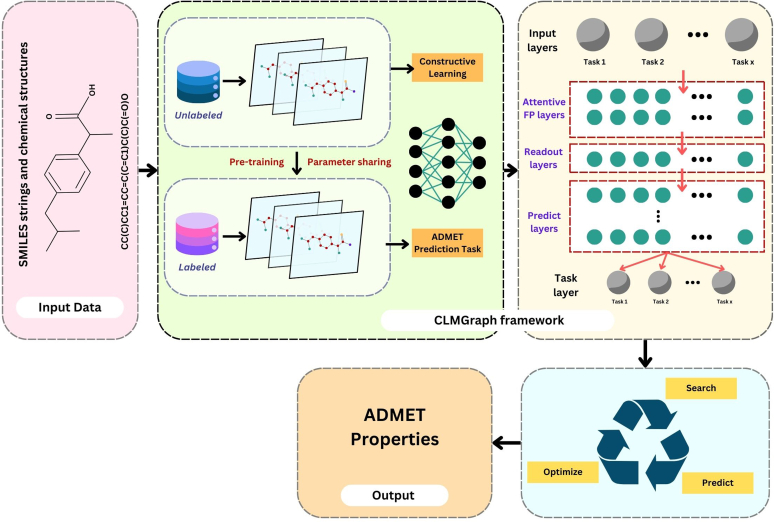
CLMGraph model architecture.

The contrastive learning-based multitask graph neural network model (CLMGraph) is a heterogeneous graph with various classifications of edges and 2 classification nodes (item and user). At first, a single representation of several nodes is generated by the customized user-specific interactions. Second, constructive learning and loss functions are computed based on the numerous nodes after it has generated its views and purpose. Third, multichannel NNs are used to generate representations of user-item interactions. Lastly, using interrelationships between various propagation layers, the higher-order multirelationships of users in the structure of interaction graphs are modeled in various embedded propagation layers (Fig. 7) in GNNs.^48^ Molecular pairs for contractive learning techniques are built using the QED value of millions of small molecules to improve the models' overall representational power during the pretraining stage. The pretraining model is then strengthened for the prediction after multitask property prediction phase.^49^ To optimize the ADMET model, Mean Squared Error Loss was used for the ADMET regression tasks and BCELoss (Binary Cross-Entropy Loss) for the classification tasks.^50^

(31) $$Mean\;Squared\;Error\;Loss=\frac{1}{N}\sum_{i=1}^{N}{\left(y_{i}-{ŷ}_{i}\right)}^{2}$$

where N denotes number of data samples in the batch, $y_{i}$ is true value of the $i^{th}$ sample, and ${ŷ}_{i}$ is the predicted value of the $i^{th}$ sample.

(32) $$\text{BCEloss}=-\frac{1}{N}\sum_{i=1}^{N}\left[y_{i}.\log\left({ŷ}_{i}\right)+\left(1-y_{i}\right).\;\log\left(1-{ŷ}_{i}\right)\right]\;$$

Herein, $y_{i}$ indicates whether the nodes are similar (1) or not (0). The integration of CLMGraph framework with ADMET prediction model enhances the robust and rapid predictions and facilitates the batch processing of ADMET property information for compounds.

#### 3.3.5 Feature-enhanced graph neural network-neural decision tree

**Fig. 8:**
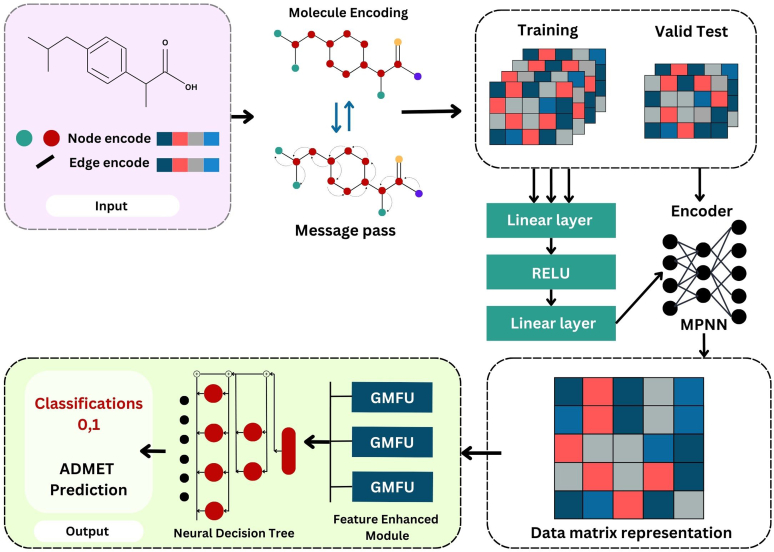
FE-GNN-NDT model architecture.

An innovative method called feature-enhanced graph neural network-neural decision tree combines a graph-based model with neural decision trees and Gate Modulation Feature Units (GMFUs) at the output layer (Fig. 8). This effectively predicts the ADMET properties and provides the high-dimensional information for the feature representation.^51^ In the input layer, SMILES are converted to molecular graphs to begin the model architecture. In graph conversion, the chemical bond is represented by edges, whereas the node is represented by multiscale properties like valency and charge. The MPNN passes the encoded molecules through for refinement and additional processing. It also equips the training and test sets with trained weights. The developers used ensemble approach to build this model for molecular classification. Particularly the final output of molecular encoding was produced by individually training several outputs of every molecule. The next approach by the developers was to further refine the task-relevant feature detection for tasks that come after. A number of GMFUs were used in the model to improve the feature selection process. The GMFU final output is directed by the update gate, which combines current candidate features with the previous hidden states. The inputs were then processed using GMFU and differentiated using neural decision trees, which functions as a differentiable binary decision tree. Soft decisions that produce continuous probabilities are used in the decision tree. Its function is to convert a k-dimensional input into an output in 2 dimensions.^52^ Furthermore, the separation between substrates and nonsubstrates is much improved with this approach, which is crucial for capturing pharmacophoric characteristics and ADMET predictions.

#### 3.3.6 An enhanced Graphormer model

**Fig. 9:**
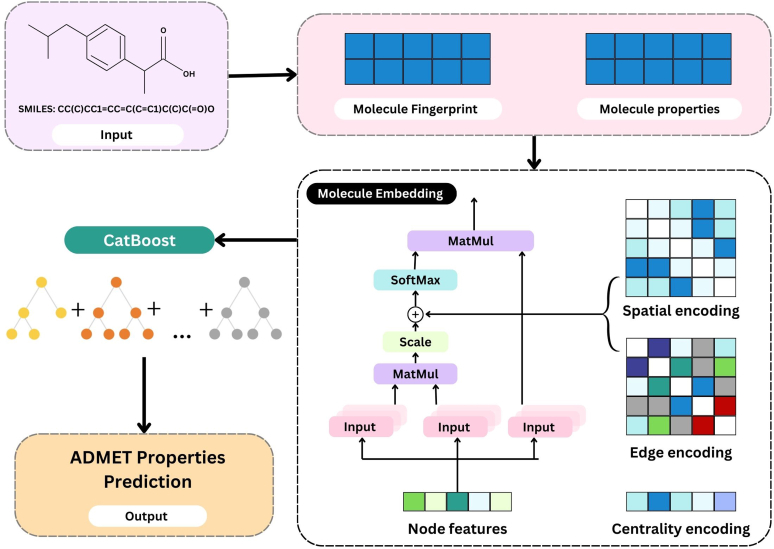
Enhanced Graphormer model architecture.

An enhanced DL model called Graphormer adapts the transformer architecture for graph-based tasks, such as link prediction, node classification, and graph classification (Fig. 9). Although they are limited to physicochemical characteristics and molecular fingerprint-like features, Graphormer models are excellent at retrieving the position and connection information of the small molecules inside the topology.^53^ In order to address these problems, enhanced Graphormer models have recently been proposed, with a tree model added for secondary training, which has increased prediction accuracy and provides deeper insights. The model architecture is primarily divided into 2 phases. The initial phase includes fine-tuning and several iterative refinements of the inputs as well as pretraining models to capture the fundamental pattern in molecular data. Specifically, CatBoost was used in the second training phase of the tree model. It aids in the thorough integration of many kinds of information, including molecularity. Lastly, the model was assessed with exacting matrices as Symmetric Mean Absolute Percentage Error, Pearson correlation coefficient, and R-square to predict the ADMET properties. Additionally, the model integrates molecular characteristics and molecular fingerprinting according to the generated embeddings. The 3 types of information contained in this include centrality, edge, and spatial encoding. The edge encoding provides the connections between nodes, centrality encoding describes the significant levels of all nodes in the graph, and spatial encoding describes the technique of expressing the spatial positions of the nodes. This enhanced Graphormer model takes into account not only the embedding features but also the physiochemical and molecular fingerprints.^54^ The ADMET properties are more accurately predicted by this combined model compared with conventional computational models.

#### 3.3.7 Transformer-based molecular graph BERT model

**Fig. 10:**
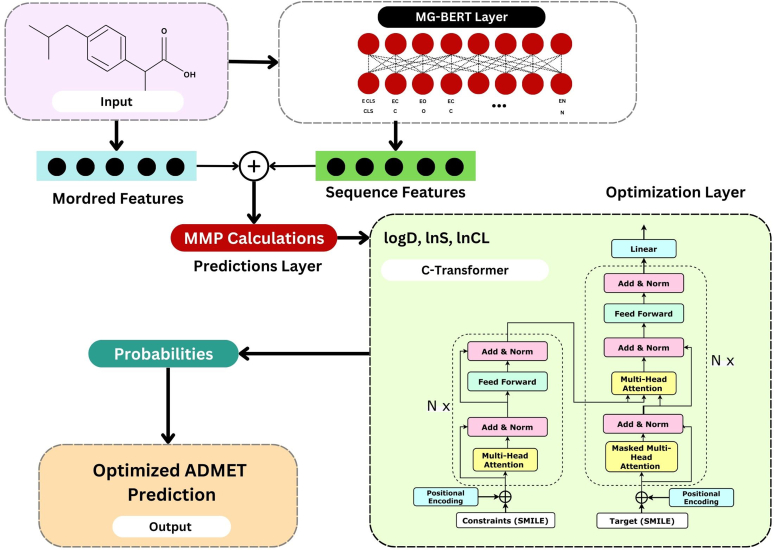
Transformer MG-BERT modelarchitecture

The molecular graph BERT model (MG-BERT) serves as the base for this ADMET prediction model, which also incorporates Mordred descriptor and molecular graph features. Although the prediction layer had both molecular graph and Mordred descriptor characteristics that were extracted and integrated from this layer, MG-BERT^55^ was utilized to extract deep features from the molecular graph (Fig. 10). In the ADMET prediction task, Mordred turned out to be the most important component. This model was later integrated with the Constraints-Transformer optimization layer. This layer is used to choose and optimize parameters like solubility (ln S) and lipophilicity (log D). The first step in the prediction procedure is to calculate the matched molecular pairs by extracting deep characteristics from the input molecules. The 2 kinds of constraints are also present. Target and input molecules changed value and discrete property values. Additionally, the chemical transformation involved in the matched molecular pair is learned using the whole transformer model. One token of small molecules and property constraints are present in this input transformer encoder. The target molecules, represented as SMILES, are the input decoder data. In order to extract the deep features of the input molecules, many encoder layers are stacked. ResNet, Add, and Norm are added and included several times in each layer to keep this deeper model from forgetting the initial features. The final prediction is obtained after the model has been trained by minimizing the Kullback–Leibler divergence loss between the model output and the decoder's input.^56^ This model has demonstrated 97% of the target molecules produced by the C-Transformer meet the structural constraints, and 59% meet both the structural and property constraints, according to the evaluation results on the test set. As a tool for molecular optimization, this transformer MG-BERT model can give chemists trustworthy direction and offer a practical approach for a coordinated optimization of PD and PK parameters.

## 4 Application of AI modeling in ADMET prediction

To develop an AI model for the prediction of ADMET of newly designed compounds, larger data training sets are required to train the model to make the predicted values more reasonable and accurate. There are a large number of reports published indicating the use of developed AI models to predict the ADMET properties of the new compounds.^57^, ^58^, ^59^, ^60^, ^61^, ^62^, ^63^, ^64^, ^65^, ^66^, ^67^, ^68^, ^69^, ^70^, ^71^, ^72^, ^73^, ^74^, ^75^, ^76^, ^77^, ^78^, ^79^, ^80^, ^81^, ^82^, ^83^, ^84^, ^85^, ^86^, ^87^, ^88^, ^89^ Tables 2 and 3 enlist the datasets and various AI applications successfully employed in the prediction of ADMET.

**Table 2:** Data sources for AI-based ADMET predictions

S.no	Source	Data Size	Website/Link
1	NIST Chemistry WebBook	>90,000	https://webbook.nist.gov/
2	GOSTAR	1.76 million	https://www.gostardb.com/
3	openFDA	>3 million	https://open.fda.gov/
4	SIDER 4.1	55,730	https://sideeffects.embl.de/
5	KEGG	12,607	https://www.kegg.jp/
6	DrugBank 5.1.9	14,665	https://www.drugbank.ca/
**7**	PubChem	>119 million	https://pubchem.ncbi.nlm.nih.gov/
8	IMPPAT 2.0	17,967	https://cb.imsc.res.in/imppat
9	ZINC20	>750 million	https://zinc20.docking.org/
10	ContaminantDB	54,249	https://contaminantdb.ca/
11	ChemSpider	115 million	https://www.chemspider.com/
12	Therapeutics Data Commons	4,264,939	https://tdcommons.ai/
13	OCHEM 4.2	4,164,779	https://ochem.eu/home/show.do
14	BindingDB	>1 million	https://www.bindingdb.org/
15	ChEMBL	>2.2 million	https://www.ebi.ac.uk/chembl/
16	TCMSP	12,144	https://tcmsp-e.com/load_intro.php?id=40
17	DILIrank	1036	https://www.fda.gov/science-research/liver-toxicity-knowledge-base-ltkb/drug-induced-liver-injury-rank-dilirank-20-dataset
18	LTKB-BD	287	https://www.fda.gov/science-research/liver-toxicity-knowledge-base-ltkb/ltkb-benchmark-dataset
19	GDB	970 million	https://www.gdb.unibe.ch/

**Table 3:** List of recently developed AI model for ADMET predictions and its applications

S.No	Modeling	Endpoint	Target	Dataset Size	Performance	Data Sources	Algorithms	Interface/Software	Description	Ref
1.	AI	Absorption	Human intestinal absorption (HIA)	1242	ACC = 90.38% PR = 91.13% Recall = 96.44% SP = 80.63% MCC = 0.80	-	SVM, ANN, kNN, PNN, PLS, and LDA	http://modem.ucsd.edu/adme/	In this research, they conducted a comparative examination of several models, and SVM outperformed all other models. The SVM-based prediction model accurately predicts whether a substance will be well or badly absorbed. Prediction models are important in early stages of medication design and development.	^57^
2.	ML	Absorption	Caco-2	5654	R^2^ = 0.076 RMSE = 0.771 MAE = 0.629 Pearson = 0.702 Spearman = 0.704	Literature	XGBoost	-	In this research study, Caco-2 permeability dataset was expanded and assessed ML techniques using various chemical representation. XGBoost outperformed equivalent models in the test sets and predictive when applied to industrial data.	^58^
3.	ML	Distribution	BBB permeability	7162	ACC = 89% SP = 0.77 Recall = 0.93	Literature	LightGBM	http://ssbio.cau.ac.kr/software/bbb (LightBBB)	Training a model with a huge dataset ensures coverage of the chemical diversity of BBB molecules, crucial for accurate predictions. Models trained with a small dataset may not accurately represent the chemical variety of compounds due to biased creation.	^59^
4.	DL	ADMET	-	2579	R^2^> 0.6 AUROC > 0.85	41 ADMET datasets from TDC	Chemprop-RDKit	https://admet.ai.greenstonebio.com/ (ADMET-AI)	ADMET-AI is an open source, free, and user-friendly platform that identifies compounds with favorable ADMET profiles for further research.	^60^
5.	ML	Absorption	HIA	141	RMSE = 17.63 NRMSE = 18.55% R^2^ = 0.379 ACC = 0.7653	ZINC and ChEMBL	Three CatBoosts, 2 NNs, 1 DT, and 1 RF	-	The AI-based system is a hierarchical collection of classification and regression models. Combining 2 models into one system expands the range of molecules classed as extremely permeable and accurate.	^61^
6.	ML	Distribution	Plasma protein binding (PPB)	100,550	RMSE = 0.444 R^2^ = 0.721	AstraZenecain-house	SVM	-	In this research study, it is focused on single in silico ADME models, multitask learning has lately emerged as a promising approach. In silico ADME models are widely accepted at AstraZeneca and used to prioritize compounds for measurement.	^62^
7.	ML	ADME	Gastrointestinal absorption, BBB Permeability	148	ACC = 98%, PR = 97%, Error rate = 2%	IMPPAT PubChem	SVM, RF, NB, and DT	http://www.swissadme.ch/index.php (SwissADME)	This study used ML techniques to accurately and efficiently predict drug-like characteristics. DTs outperformed others.	^63^
8.	DL	Absorption	log P	14,050	RMSE = 0.47	PHYSPROP, SAMPL6	DNN	-	DNN models can improve log P dataset curation by spotting errors and addressing restrictions.	^64^
9.	DL	Absorption	log P	14,176	RMSE = 0.62	PHYSPROP	DNN	-	In this study, deep neural network and chemical fingerprints were used to build a unique method for predicting the logarithm of partition coefficient (logP).	^65^
10.	ML	Absorption	log P	11	RMSE = 0.34	SAMPL6	D-MPNNs	-	D-MPNN-based logP prediction model outperformed other approaches in analyzing substances from the last SAMPL6 challenges.	^66^
				22	RMSE = 0.66	SAMPL7				
11.	ML	Absorption	log P	707	R^2^ = 0.71 RMSE = 0.91	ZINC database	FElogPmodel	-	FElogP outperformed other logP models, including QSPR, machine learning, and continuum solvation models.	^67^
12.	DL	Absorption	Log P	13,821	R2 = 0.925	ChEMBL, PubChem, DrugBank, literature	GCNN, GAT	http://cadd.pharmacy.nankai.edu.cn/interpretableadmet/ (Interpretable-ADMET)	Interpretable-ADMETs provide a optimize module to generate new virtual candidates based on matched molecular pair rules. It is believed that interpretable-ADMET can be a beneficial tool for optimizing pharmacological leads.	^68^
			Log S	9603	R2 = 0.785					
			Caco-2	1442	ACC = 0.826 SP = 0.736 MCC = 0.628					
			HIA	863	ACC = 0.993 SP = 1.000 MCC = 0.970					
		Distribution	pgp-inhibitor	1738	ACC = 0.932 SP = 0.945 MCC = 0.853					
			pgp-substrate	1087	ACC = 0.840 SP = 0.888 MCC = 0.672					
		Metabolism	CYP1A2	12,805	ACC =0.814 SP = 0.777 MCC = 0.625					
			CYP2C19	12,905	ACC = 0.790 SP = 0.777 MCC = 0.578					
		Excretion	T_1/2_	665	ACC = 0.773 SP = 0.877 MCC = 0.553					
		Toxicity	hERG	8672	ACC =0.919 SP = 0.965 MCC = 0.612					
			Carcinogenicity	1146	ACC =0.716 SP = 0.787 MCC = 0.387					
13.	DL	Absorption	Caco-2	2464	R^2^= 0.786 RMSE = 0.296 MAE = 0.203	ChEMBL, PubChem, OCHEM, literature	MGAF	https://admetmesh.scbdd.com/ (ADMETlab 2.0)	The MGA framework's robust QSPR models provide more accurate predictions than typical ML algorithms.	^43^
			MDCK	1140	R^2^ = 0.662, RMSE = 0.301 MAE = 0.233					
			HIA	1160	ACC = 0.949 SP = 0.867 MCC = 0.785					
		Distribution	PPB	4712	R^2^= 0.744 RMSE = 0.155 MAE = 0.091					
			BBB	2865	ACC = 0.852 SP = 0.810 MCC = 0.698					
		Metabolism	CYP 1A2	12,635	ACC = 0.886 SP = 0.876 MCC = 0.771					
			CYP 2C19	366	ACC = 0.854 SP = 0.889 MCC = 0.712					
		Excretion	T1/2	1219	ACC = 0.744 SP = 0.750 MCC = 0.481					
			CL	831	R^2^ = 0.692 RMSE = 2.956 MAE = 1.883					
		Toxicity	hERG	13,845	ACC = 0.889 SP = 0.866 MCC = 0.778					
			Carcinogenicity	1041	ACC = 0.619 SP = 0.566 MCC = 0.240					
14.	DL	Absorption	LogP and permeability	13,000	R^2^ = 0.892 MSE = 0.359	OPERA	DNN	-	Lipophilicity description DeepFL-LogP is based on DNN. DeepFL-LogP has a strong association with experimental partition coefficient reference data for drug-like compounds.	^69^
15.	AI/LLM	Distribution	BBB permeability	12,452	AUC = 0.90	B3DB, Zinc-15, and NPRL of CMUH datasets	Mega MolBART, XGBoost	https://catalog.ngc.nvidia.com/orgs/nvidia/teams/clara/models/megamolbart (NVIDIA, Version 1.0)	This research highlights the advantages of large language models, such as MegaMolBART, over traditional ML approaches. SMILES molecular representations can accurately estimate BBB permeability, eliminating the need for extra physicochemical property computations	^70^
16.	ML	Absorption	HIA	1516	BACC = 0.84 AUC = 0.89	OCHEM	RF	https://gitlab.com/vishsoft/fpadmet FP-ADMET	Fingerprint-based ML algorithms effectively predict a wide range of ADMET features. Fingerprint-based RF models outperform traditional 2D/3D molecular descriptors in most cases.	^71^
			Oral bioavailability	1822	BACC = 0.71 AUC = 0.77					
			MDCK	701	R^2^ = 0.62 RMSE = 0.61 MAE = 0.44					
		Distribution	PPB	8103	BACC = 0.82 AUC = 0.92					
			Human placenta barrier	88	R^2^ = 0.41 RMSE = 0.24 MAE = 0.20	Literature				
		Metabolism	CYP450 (1A2) inhibition	17119	BACC = 0.85 AUC = 0.93	Literature				
			BCRP inhibition	2799	BACC = 0.89 AUC = 0.95	Literature				
		Excretion	Intrinsic clearance (CLint)	244	R^2^ = 0.48 RMSE = 0.83 MAE = 0.65	Literature				
			Renal clearance (CLr)	636	R^2^ = 0.25 RMSE = 0.54 MAE = 0.43	Literature				
		Toxicity	AMES	7950	BACC = 0.79 AUC = 0.86	Literature				
			HepG2	6081	BACC = 0.78 AUC = 0.85	Literature				
17.	ML/DL	Distribution	BBB permeability	940	ACC = 0.96 F1 score = 0.95	Literature	SVM and GCNN	-	The combined ML and DL models were developed to provide high interpretable prediction results in CNS drug classification. Those models outperformed previous benchmark QSAR studies with high accuracy.	^72^
18.	ML	Absorption	HIA	2648	ACC = 87.71 %	OCHEM, Literature	AdaBoost, RF, XGBoost, LightGBM, LR	-	In this study, 2 DL models, a GCNN and a GAT, were trained on the same dataset to enhance predictability with automated characteristics.	^73^
19.	DL	Distribution	PPB	3921	R^2^ = 0.841 RMSE = 0.112	ChEMBL DrugBank Votano’s data set Zhu’s data set	Attentive finger print algorithm (GNN)	https://github.com/Louchaofeng/IDL-PPBopt (DL-PPBopt)	Researchers developed IDL-PPBopt, a computational solution that uses interpretable deep learning to predict and optimize PPB properties. This technique identifies substructure patterns in lead compounds and develops unique structural modification schemes using second-level chemical rules to achieve favorable PPB properties.	^74^
20.	ML	Distribution	Fraction unbound in plasma	5471	R^2^ = 0.85 RMSE = 8.44	ChEMBL	AutoML Frame work	-	AutoML toolkits can assist biological researchers without machine learning experience in creating accurate prediction models. Our prediction model for fraction unbound in plasma outperforms earlier models, making it useful for pharmacokinetic modeling, in silico drug design, and discovery.	^75^
21.	ML	Distribution	Volume of distribution (VDs)	1303	GMFE = 2.15% < 2-fold = 54% < 3-fold = 73	Literature	RF	-	The novel RF approach, using only 6 descriptors, enables scientists to develop molecules with desired human VD values. In silico VDs models function well on structurally new compounds, indicating great confidence in employing them for compound design and dose prediction.	^76^
22.	DL	Distribution	BBB permeability	2350	ACC = 96.53% SP = 98.08%	Literature	RNN	-	The RNN-BBB model successfully solved all concerns, confirming the validity of the triple constraints and recommended solutions. This model outperformed previous studies in the literature.	^77^
23.	ML	Absorption	Skin permeability (LogK p)	2326	R^2^= 0.819 RMSE = 0.437 MAE = 0.278	Drug Bank	LGBM	-	This study proved, AI algorithms can accurately estimate skin permeability for chemicals. Boosting methods, particularly LGBM, outperformed other models in predicting LogK p, resulting in strong quantitative estimation.	^78^
24	AI/ML	Absorption	Aqueous solubility	12,054	ACC = 75% MCC = 0.4	BioPrint	RF, SVM, NB, GBM	https://www.eurofinsdiscovery.com/solution/safire (SAFIRE platform)	The SAFIRE models, trained with high-quality data from the BioPrint database, outperformed other efforts in the field in terms of accuracy and MCC.	^79^
			Caco-2	1591						
		Distribution	PPB	5547						
		Metabolism	Metabolic stability	1720						
			CYP isoforms	521–16,054						
		Excretion	Efflux ratio	1867						
		Toxicity	hERG	27,722						
25.	AI/DL	Absorption	Membrane permeability	16,845	-	ChEMBL	NA	https://www.knime.com/ KNIME (ver. 5.2)	To construct in silico models for estimating Papp utilizing MDCK, LLC-PK1, and RRCK cell lines significant, in vitro data collection required. High-quality datasets are essential for training in silico models that predict pharmacokinetic features using AI and DL.	^80^
26.	AI/ML	Absorption	Permeability	2490	Efficiency = 0.59	Janssen compounds in house dataset	GCNN	-	Janssen's in house ML model, gTPP, can predict 18 ADME end points. Chemprop, a graph CNN method, does well in predicting chemical properties.	^81^
		Distribution	PPB	627	Efficiency = 0.59					
		Metabolism	1A2	4080	Efficiency = 0.68					
			2C8	4151	Efficiency = 0.55					
			2C9	4146	Efficiency = 0.60					
		Excretion	Efflux ratio	2384	Efficiency = 0.35					
27.	ML	Toxicity	Hemolytic toxicity	805	R^2^ = 0.653	Hemolytic toxicity dataset (Dataset-Whole)	kNN, SVM, RF, GBM	-	The endpoint has never been quantitatively predicted due to lack of adequate datasets. In this study the dataset is manually collected containing small molecules from a variety of literature. Based on the dataset researcher developed first ML-based regression model to predict hemolytic toxicity.	^82^
28.	ML	Toxicity	Mitochondrial toxicity	3407	AUC = 0.899 AUC = 0.921	PubChem	kNN, LR, RF, SVM, XGBoost	-	In this study, 45 binary classification models were created to predict chemical mitochondrial toxicity utilizing 9 molecular fingerprints and 5 machine learning algorithms. The parameters of 5 algorithms were then adjusted using ten 10-fold cross validations, and the prediction models were evaluated by the test set.	^83^
29.	DL/ML	Toxicity	NR-AhR	6549	AUC = 0.830	Molecule Net	SSL-GCNN	https://app.cbbio.online/ssl-gcn/home (Tox21)	SSL-GCN models outperform the built-in DeepChem ML algorithms. This research showed SSL can improve model prediction accuracy by learning from annotated data.	^84^
			NR-AR-LBD	6758	AUC = 0.764					
			NR-AR	7265	AUC = 0.796					
			NR-PPAR-gamma	6450	AUC = 0.761					
30.	DL	Toxicity	Hematotoxicity	2383	AUC = 0.768	SIDER, OCHEM, PubChem Bioassay	GCNN, DMPNN	-	In this study dataset of 2383 hematotoxic and nonhematotoxic compounds were collected and built classification models with 7 ML algorithms and 9 chemical representations. Among these, the attentive FP classifier performed well.	^85^
31.	ML/DL	Toxicity	Carcinogenicity	2355	AUC = 0.76 ACC = 0.81 F1_score = 0.20 MCC = 0.15	PharmaPendium, ChemIDplu, literatures	DMPNN	-	This study demonstrated DL-based D-MPNN technique was generally superior than the standard ML models, and good performance was observed for the majority of human organ level toxicity end points in this investigation.	^86^
			Cardiotoxicity		AUC = 0.80 ACC = 0.73 F1_score = 0.72 MCC = 0.72					
			Developmental toxicity		AUC = 0.80 ACC = 0.71 F1_score = 0.70 MCC = 0.45					
			Hepatotoxicity		AUC = 0.78 ACC = 0.71 F1_score = 0.61 MCC = 0.39					
32.	ML	Metabolism	CYP1A2, CYP2C19, CYP2D6, CYP2C9, and CYP3A4 inhibitors	18,313	ACC = 0.97 AUC = 0.98	PubChem SuperCYP	RF	http://insilico-cyp.charite.de/SuperCYPsPred/ (SuperCYPsPred)	The SuperCYPsPred web server presently focuses on 5 key CYP isoenzymes: CYP1A2, CYP2C19, CYP2D6, CYP2C9, and CYP3A4, which account for about 80% of clinical drug metabolism.	^87^
33.	ML	Metabolism	CYP450	134,844	AUC = 0.92 ACC = 0.83	ChEMBL, PubChem, ADME	RF	https://nerdd.univie.ac.at/cyplebrity/ (CYPlebrity)	Researchers developed ML models to classify small compounds as inhibitors or noninhibitors of 5 CYP isozymes that are important in human xenobiotic metabolism. RF classifiers are trained using large collections of known inhibitors and noninhibitors. Combining this data enhanced the prediction of enzyme toxicity.	^88^
34.	DL	Excretion	Clearance (CL)	139,907	R^2^ = 0.59 RMSE = 0.35	AstraZeneca in house	MT-CNN	-	Four regularly researched clearance endpoints were chosen from AstraZeneca internal sources, giving a solid foundation for creating multitask CNN models. CNN models successfully captured implicit chemical correlations in training and test data, identical to structural fingerprints.	^89^

ADMET, Absorption, Distribution, Metabolism, Excretion, and Toxicity; AUROC, area under the receiver operating characteristic curve; BACC, balanced accuracy; CNS, central nervous system; DMPNN, Directed Message Passing Neural Network; DT, Decision Tree; FP, fingerprint; GAT, graph attention network; LDA, Linear Discriminant Analysis; LGBM, Light Gradient Boosting Machine; LR, logistic regression; MAE, mean absolute error; MDCK, Madin-Darby canine kidney; MGAF, multi-granularity attention fusion; NB, Naïve Bayes; NRMSE, normalized root mean squared error; RMSE, root mean square error; RRCK, Ralph Russ canine kidney; SSL, semi supervised learning; TDC, Therapeutics Data Commons.

### 4.1 AI in drug absorption prediction

Drug absorption is a key physicochemical aspect of the drug design and development process, warranting special attention. Several AI models are utilized to predict the absorption properties of the new therapeutic molecule. Practical computational approaches are a must to predict the PK of the compound first and before selection and synthesis to avoid attrition during preclinical/clinical testing in the drug development process. These AI models help us understand the PK of the drug molecules, their entry into the bloodstream, and how this absorption property affects their efficacy and safety.^90^ For instance, Kumar et al^57^ demonstrated the prediction of human intestinal absorption of the compound using 6 AI models. Six prediction models were constructed utilizing 745 trial compounds to evaluate if a chemical molecule is highly or poorly absorbed. The established prediction models utilize partial least squares, k-NN, SVM, ANN, discriminant analysis, and probabilistic neural network algorithms. Test set consisting of 497 chemical compounds was used to assess prediction model accuracy. Statistical metrics, including sensitivity, specificity, precision, accuracy, and Matthews correlation coefficient, were used to determine the efficiency of constructed models based on the 4 quantitative variables. SVM surpassed the other 5 AI algorithms for the dataset in terms of prediction accuracy, with a prediction accuracy of 91.13%. The SVM-based prediction model accurately predicts whether a substance will be adequately or poorly absorbed. Prediction models are considered important in the early stages of medication design and development.^91^

Moreover, Bennett-Lenane et al^91^ conducted a comparative study on ML algorithms (SVM and ANN) to predict food impacts on bioavailability. These 2 algorithms accurately predict the food effect category of the medications approved between 2016 and 2020, of which 43% demonstrated significant food effects. This algorithm outperformed food effects predictions using Biopharmaceutics Classification System and older drug datasets. Predictive modeling discovered critical physicochemical characteristics influencing drug absorption, including topological polar surface area, hydrogen bond donors, S+logP (partition coefficient), and dose. ANN employed in this work can quickly screen drug candidates for potential food effects during the early stages of development by focusing on easily identifiable pharmacological properties.^90^ Similarly, Wang et al^58^ studied Caco-2 permeability prediction using ML algorithms. Caco-2 permeability is linked to various physicochemical properties of molecules, including hydrogen bonding, lipophilicity, ionization, and size. These criteria can help forecast Caco-2 and guide medicinal chemists in optimizing lead compounds. Caco-2 permeability is important for improving the efficiency of oral medication development.^58^

### 4.2 AI in drug distribution prediction

Predicting drug distribution properties is a critical component of PK, which is important for comprehending how drugs travel through the body and reach their intended targets. It also plays a significant role in predicting a compound's toxicity and efficacy.^92^ AI-based models have demonstrated remarkable potential in predicting key properties such as BBB permeability, plasma protein binding, and volume of distribution. These models leverage various ML and DL techniques.^93^ For instance, Shaker et al^59^ studied the prediction of BBB permeability using the LightBBB server model. This model illustrates computational PK studies utilizing Light GBM algorithms on a dataset of 7162 compounds, achieving an impressive accuracy of 89% and an area under the time-concentration curve (AUC) of 93%. Another study on the same dataset performed even better with the help of a mixed DL model with an ACC of 92% and an AUC of 96%. The results demonstrate how AI may be used to predict BBB permeability, which is essential for drug discovery and central nervous system medication design.^59^ Another study performed by Iwata et al involved developing a ML-mediated multimodal method for prediction of total drug clearance and volumes of distribution by imputing various nonclinical data. The model for total body clearance achieved a performance metric with a geometric mean fold error of 1.92 and percentage within 2-fold error of 66.5% and for the steady-state volume of distribution model, the geometric mean fold error was 1.64 with 2-fold error percentage of 71.1%. The result indicated that the models are competitive with traditional animal scale-up methods that often require extensive animal testing.^94^

### 4.3 AI in drug metabolism prediction

AI advanced models can now integrate vast amounts of data, allowing for more renounced predictions regarding how drugs are metabolized in the body. The future of AI in drug metabolism prediction has a promising impact, with ongoing advancements in ML and data analytics. These advancements are expected to enhance predictions’ precision further and streamline the drug development process.^95^ For instance, Nigam et al^96^ addressed liver or kidney disease by the organic anion transporters and organic anion transporting polypeptides (OATPs) specificity by employing the ML and DL classifications using 80 drugs. They identified 8 physiological properties that indicated a high tendency for drug interactions when associated with liver and kidney transporters. Liver OATPs preferred drugs with higher hydrophobicity and complexity, whereas kidney OATPs preferred more polar drugs. Various ML and DL models were also developed to predict new OATP1B1 inhibitors and were validated experimentally.^96^ Moreover, Jang et al^97^ developed an ML model called as PK-drug-drug interaction prediction, using a reliable database of 3627 drug-drug interactions derived from 38,711 FDA drug labels. This study focused on AUC fold change. It achieved root mean squared error of 0.5959 signifying that its predictions closely approximate the actual values. The evaluations of forecasts were based on their adherence to specific ranges of fold change, with 75.77% of predictions falling within 0.8- to 1.25-fold, 86.68% within 0.67- to 1.5-fold, and 94.76% within 0.5- to 2-fold. Most predictions were rated as “good” or “moderate.”^97^

### 4.4 AI in drug excretion prediction

By simulating the biological processes, the in silico AI models can estimate the drug excretion parameters like the fraction of drug excreted unchanged in urine (f_e_) and renal clearance (CLᵣ). AI models assist by identifying well-excreted drugs, reducing toxicity concerns. These models also reduce the developing cost and time by analyzing large datasets and predicting drug behavior. The AI-driven techniques increase research accessibility and promote productivity and creativity in drug discovery.^98^ For instance, Watanabe et al^99^ studied datasets of 401 and 411 compounds using an open source tool and developed in silico models to predict the f_e_ and CLᵣ using chemical structure data. The classification model for f_e_ achieved a balanced accuracy of 0.74, whereas the CLᵣ prediction system enhanced when integrating plasma protein binding data, with 78.6% accuracy for higher clearance compounds within a 2-fold error. The analytical result showed that less lipophilic drugs tend to be renally excreted, highlighting overlapping chemical spaces among excretion types. The model is a valuable tool for medicinal chemists in early drug discovery.^99^ Similarly, Jung et al^100^ used ML model DNN to explain drug excretion. The concatenation model (Concat) integrates SMILES representations with physicochemical properties to improve predictions concerning drug excretion, yielding an area under the receiver operating characteristic curve of 74.9%. By synergistically integrating molecular architectures with chemical attributes, it substantially elevates predictive accuracy compared with models reliant on singular sources. Then, the incorporation of ChemBERT further fortifies the representation of molecular entities.^100^

### 4.5 AI in drug toxicity prediction

In medicinal chemistry and pharmacology, accurately predicting chemical toxicity is a difficult task, as it is linked to drug developments effectiveness and safety. Toxicity assessment is crucial in drug development as it significantly impacts candidate attrition rates. AI improves drug toxicity prediction by identifying potential harmful effects of novel compounds before human clinical trials.^101^ For instance, Yuan Li et al.^102^ developed 3 models (Des MT-DNN, FP MT-DNN, and MT-EGCN) for single and multitasking using 2D and 3D descriptors, molecular graphs, and fingerprints. The models were validated with benchmark tests on the Tox21 data challenge. Multitask learning effectively addresses the imbalance problem in Tox21 datasets and improves prediction performance compared with single-task approaches. To improve prediction accuracy, the researchers developed a comodel that uses averaging to balance the impact of several molecular representations and techniques. Comodel outperforms current research in classification performance, with ACC 0.952, AUC 0.903, F1 0.573, BAC 0.725, precision 0.814, kappa 0.549, and Matthews correlation coefficient 0.580, on the test set.^102^ Similarly, Fradkin et al^103^ demonstrated carcinogenicity prediction using a DL model called CONCERTO, which uses a graph transformer and molecular fingerprint representation to predict carcinogenicity based on molecular structure. Extensive studies concluded that this model performs effectively and can be used for external validation sets. CONCERTO potentially identifies known toxicophores and can guide future carcinogenicity research and shed light on the molecular underpinnings of carcinogenesis.^103^

## 5 Discussion

Increasingly diverse data sources and modeling methodologies in PK, drug metabolism, and toxicokinetic research are reflected in the increasing number of AI-driven models for ADMET prediction. Table 3 summarizes the key ADMET models that have been established recently, together with comparison analyses that provide a thorough understanding of their relative advantages and limitations. From the perspective of data availability and requirements, ML models like RF, SVM, and GBM operate best on moderately sized, structured datasets (10^3^–10^4^ samples). In contrast, DL and transformer-based models like RNNs, GCNNs, and MegaMolBART require large datasets (≥10^4^ compounds) in order to extract meaningful features. High-quality labeled data is still a significant barrier, nevertheless, particularly when it comes to hematotoxicity, such as rare ADMET endpoint predictions. Subsequently, the ML models are simpler to understand, enabling users to link predictions to molecular descriptors and comprehend the factors that influence ADMET outcomes. On the other hand, limited interpretability is a problem for the DL and graph-based models. By connecting learned features to interpretable substructures or rules, a number of ADMET models, including Interpretable-ADMET and IDL-PPBopt, are utilized hybrid and attention-based architectures to address this problem and which enhances the model’s performance. Additionally, from the perspective of computational cost and infrastructure, DL needed GPU-enabled infrastructure for model training and optimization, whereas ML algorithms may be executed efficiently under regular operating settings. NNs based on transformers and graphs are computationally expensive; however, cloud-based systems (such NVIDIA Clara, ADMET-AI, and ADMETlab 2.0) have made these models more widely available via web interfaces.

Finally, for the absorption predictions such as logP/logS, Caco-2, and human intestinal absorption both DL/ML models are produced comparable accuracy ranging upto R^2^ 0.9. This results due to the both models are capturing structures as well as topological information. For the distribution prediction including BBB, volume of distribution, and plasma protein binding, the hybrid and DL models are fit for this especially the models like RNN and GCNN which give the better results than the other models. For the metabolism like CYP450 predictions ML models are very well fitted due to tabular descriptions which continuously give the robust results with several ML models including RF and XGboost. For the toxicity and excretion prediction hybrid models with DL architectures give the better results due to the multiple task algorithms increased the prediction accuracy of carcinogenicity, hERG predictions and increased the generalization for the t_1/2_ and clearance. Overall, the hybrid models required large and high-dimensional data with heterogeneous biological data such as omics, chemical descriptors like SMILES, text-based data, and biological network data which improved the accuracy of the ADMET predictions. Increasing this advanced data integrating with hybrid DL/ML models overcomes the problems facing with the traditional DL and ML models and increasing the scalability and accuracy when trained on sufficiently large and diverse datasets. Moreover, Table 4 gives the comparative analysis of these AI models and its pros and cons which gives the evident that such models are given the solid foundation for the ADMET predictions.

**Table 4:** Comparative summary of this AI model classes for ADMET prediction.

Model Class	Performance Trend (ADMET) and Data Requirement	Interpretability	Talent Requirement	Computational Cost and Infrastructure Need	Representative Tools/Models	Pros	Cons
ML	Best for AM Small-moderate (10^3^–10^4^)	High (descriptor based)	Basic coding/statistics required	Low cost Standard workstation required	SwissADME, FP-ADMET, SuperCYPsPred	• Low resource demand • Performs well with limited data • Easy to train and interpret	• Weaker on unstructured or multimodal data • Limited scalability • Limited ability to model complex nonlinear relationships
DL	Best for ED Large (≥10^4^)	Low-moderate	Intermediate to advanced specializations required	High cost and GPU/cloud required	ADMET-AI, ADMETlab 2.0, DeepFL-LogP	• Learns complex nonlinear patterns • Excellent at capturing nonlinear relationships • Highly scalable to large datasets	• Requires large labeled datasets • Overfitting risk • Low interpretability
Hybrid ML-DL	Promising for all ADMET Medium to large (≥10^4^)	Moderate	Advanced specializations required	High cost and GPU/cloud required	XGBoost + CNN, multitask networks	• Flexible for multimodal data • Combines interpretability and feature learning • More flexible and robust across diverse datasets	• Integration complexity • Requires expertise in multiple algorithms • Tuning and deployment more challenging
Transformer and LLM-based Models	Promising for all ADMET Very large (≥10^5^)	Low-moderate	Advanced AI/ML specialization required	Very high cost and GPU/cloud required	MegaMolBART (NVIDIA Clara), ChemBERTa	• Handles heterogeneous data • Excellent generalization	• Difficult to fine-tune • Limited transparency • Data and resource intensive
Graph-based models	Best for BBB, PPB, toxicity Large (≥10^4^)	Moderate-high (interpretable substructures)	Advanced chemoinformatics with coding specialization required	High cost and GPU/cloud required	Interpretable-ADMET, IDL-PPBopt	• High accuracy in structure-dependent tasks • Captures molecular topology	• Sensitive to graph representation quality • High computational cost

## 6 Challenges and future directions

The scalability and prediction accuracy of ADMET predictions have been improved using AI-driven models. However, it can still be challenging in scenarios when prediction models are needed on a larger scale in businesses that need to generate meta data to link several different datasets. The use of AI techniques would then be made possible by an end to end data link and flow. The scientific community can benefit from AI-driven ADMET prediction services to some extent, but the data used limits the underlying models. The need for verification or validation for AI models is required for large-scale drug discoveries, even when the data were acquired early for prediction and annotated well. Extracting molecular structures recorded in articles, patents, or reports is another critical data curation problem. Often, the molecules are described using different combinatorial techniques, such as R-groups, and then reassembled into molecules. A technology-enabled industry requires the proper infrastructure to include AI and ML in addition to present data or systems. However, a scarcity of staff knowledgeable in these computational techniques has contributed to the poor pace of implementation.

Furthermore, advancements in the prediction process are being enhanced by future developments in AI-driven ADMET predictions, which include feature enrichment based on physical/chemical principles, the integration of 3D structural information, and sophistication through graph-based learning models. Researchers can explore these initiatives and enhance AI modeling. The potential of AI-driven technologies is starting to be recognized by regulatory bodies.^104^ By working together, regulatory agencies and AI developers can develop standardized guidelines for using AI models in drug development and ADMET predictions, speeding up the approval and market access of novel therapies. The gap between experimental research and computational modeling will also be bridged by training lab scientists and research directors on the precise data needs and quality standards for AI/ML-based studies. Building high-quality, “AI-ready” datasets that enable scientific dependability and regulatory acceptance hence requires collaborative frameworks that combine wet-lab skills with data science. Similarly, the quality and standardization of the experiment data utilized for the AI/ML models provide additional challenges beyond those related to algorithms or regulations. The models, computational complexity, the quality of the generation, curation, and dissemination of experimental data all influence the ADMET prediction outcomes. Maintaining a high standard laboratory setup that supports several institutions would increase consistency and enable reproducible and transferable data-driven models, which can be achieved with appropriate, detailed documentation and metadata storage. The future attention will be on AI-driven ADMET prediction, which may eventually lead to the next generation of AI-driven innovations. AI integration with other disciplines, tailored explicitly for customized medicine, and continuous learning will also be considered.

## 7 Conclusion

It is essential to consider whether the current developments in DL and AI are merely the next big trend or a sign of more incredible things to come. So far, the benefits of DL by itself might not be significant, but their combined use with other ML techniques might have a more substantial impact. In conclusion, our review provides fresh perspectives on hybrid learning ADMET endpoint prediction, highlighting that the pharmaceutical sector is already embracing AI, as evidenced by its partnerships and investments, as well as industry conferences and reviews. The next generation of AI-driven innovations may ultimately emerge from integrating status theory and maximum flow techniques into the architecture of ML-DL hybrid models. These techniques would enhance the identification of optimal auxiliary tasks and AI-driven ADMET predictions, including feature enrichment based on physical/chemical principles, integrating 3D structural information, and sophistication through graph-based learning models.

## Conflict of interest

The authors declare no conflicts of interest.
